# Aging of classical monocyte subsets

**DOI:** 10.18632/aging.204493

**Published:** 2023-01-16

**Authors:** Helen S. Goodridge

**Affiliations:** 1 Board of Governors Regenerative Medicine Institute, Cedars-Sinai Medical Center, Los Angeles, CA 90048, USA; 2 Department of Biomedical Sciences, Research Division of Immunology, Cedars-Sinai Medical Center, Los Angeles, CA 90048, USA

**Keywords:** aging, monocytes, myeloid progenitors

Aging impairs the function of monocytes and their progeny (macrophages and monocyte-derived dendritic cells, moDCs), which become less effective at phagocytosis and bacterial killing and produce higher basal levels of inflammatory cytokines [1–3]. We previously demonstrated that classical monocytes in mouse bone marrow comprise multiple subsets that arise independently from granulocyte-monocyte progenitors (GMPs) or monocyte-dendritic cell progenitors (MDPs) [4] (Figure 1A). In our latest study [5], we evaluated how aging impacts classical monocyte heterogeneity and gene expression in both male and female mice.

**Figure 1:**
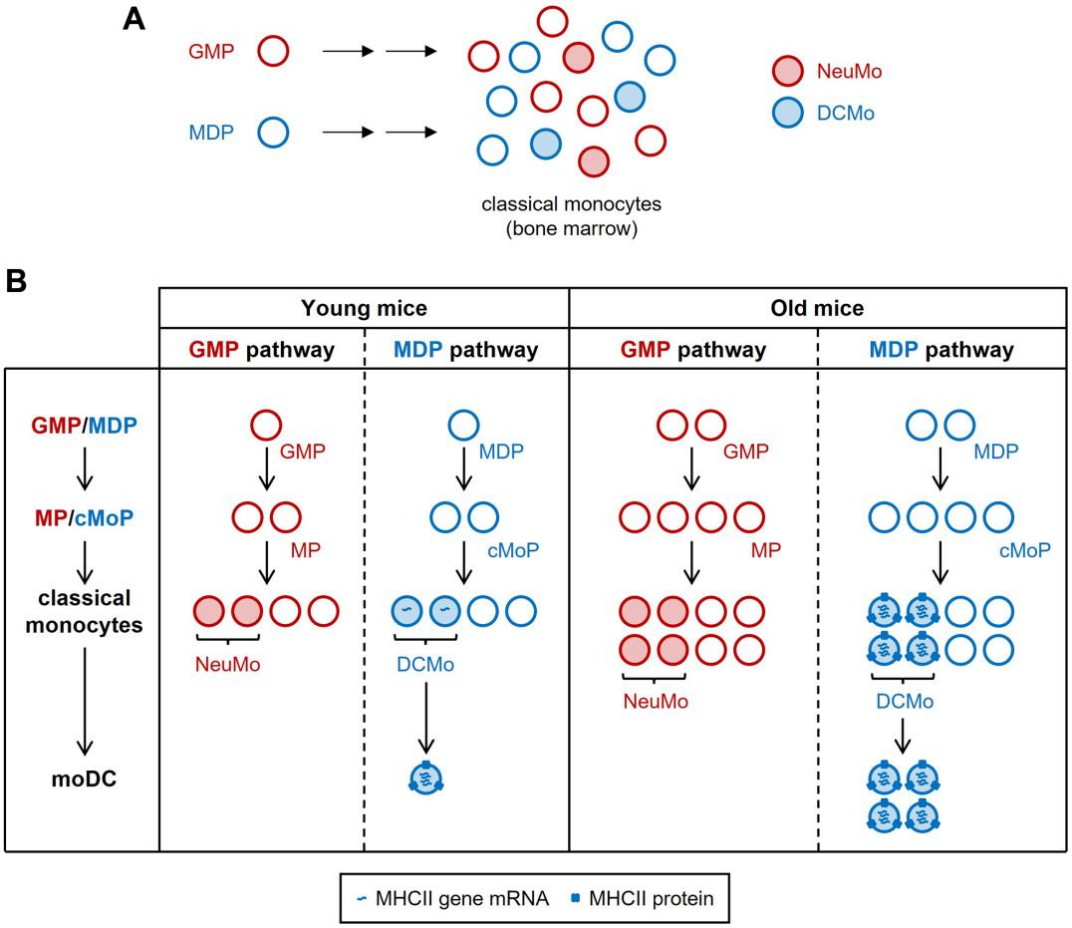
**Increased MHCII expression by DCMo during aging.** (**A**) Classical monocyte subsets arise from GMPs and MDPs, which contribute to classical monocyte heterogeneity. Neutrophil-like monocytes (NeuMo) are GMP-derived, whereas DC-like monocytes (DCMo), which give rise to moDC, are MDP-derived. The origins of other classical monocyte subsets have not yet been defined. (**B**) During aging, DCMo expression of MHCII genes increases, MHCII proteins become detectable at the cell surface, and MDPs/DCMo produce proportionally more moDC.

We used scRNAseq to define aging-associated differentially expressed genes in classical monocytes from mouse bone marrow, focusing on genes that are induced or suppressed during aging in both males and females. Strikingly, old monocytes had higher levels of transcripts encoding both major histocompatibility complex (MHC) class I (α chain) and MHC class II (α and β chains), as well as related proteins including β2-microglobulin (associates with the MHCI α chain to form the MHCI heterodimer) and CD74 (chaperones MHCII to the lysosomal pathway and prevents premature antigen binding). To validate these findings, we used flow cytometry to evaluate surface expression of MHCII and CD74 proteins. In young mice of both sexes, MHCII and CD74 expression were detectable on <5-10% of bone marrow classical monocytes, but both the proportions and numbers of MHCII^+^ and CD74^+^ classical monocytes were higher in old mice. We also detected more MHCII^+^ and CD74^+^ classical monocytes in the blood and spleen of old mice, and increased HLA-DR (MHCII) expression by classical monocytes from human peripheral blood.

These observations suggested increased output by the MDP pathway, which yields moDC via DC-like monocytes (DCMo) [4]. We identified the DCMo cluster in our scRNAseq dataset, verified that MHCII gene (*H2-Aa*, *H2-Ab1*, *H2-Eb1*) and *Cd74* expression was primarily restricted to this subset in both young and old mice, and attributed the more abundant transcripts in old monocytes to both increased expression among DCMo and a larger proportion of DCMo expressing detectable levels of these transcripts [5] (summarized in Figure 1B). In contrast, MHCI gene (*H2-K1*, *H2-Q7*) and *B2m* expression increased in all classical monocyte clusters upon aging. Moreover, expression of genes characteristic of neutrophil-like monocytes (NeuMo) produced via the GMP pathway did not change and the relative abundance of this and other classical monocyte subsets remained similar during aging, indicating a selective impact on gene expression by MDP-derived DCMo. Old monocytes also had elevated expression of the lncRNA *Aw112010* and disruption of its expression in macrophages implicated *Aw112010* in promotion of MHCII surface expression.

One prediction of these findings is that MDPs from old bone marrow would yield more moDC. We therefore cultured MDPs with GM-CSF and found that old MDPs yielded proportionally more moDC than their young counterparts. Total classical monocytes sorted from old bone marrow also yielded more moDC.

Thus, aging selectively induces transcriptomic changes in the MDP-derived DCMo subset of classical monocytes. Other classical monocyte subsets may also be altered (we did not assess gene expression changes for each subset), but the impact on DCMo appears to be particularly notable. In this study, we focused on aging changes that are common to both males and females, but we are evaluating sex differences in monocyte aging in our ongoing studies.

Importantly, our study evaluated monocytes in the basal state, and further investigation is necessary to determine how these changes shape their functional responsiveness, but it seems unlikely that increased MHC expression would enhance the capacity of monocytes or moDC for antigen presentation given that overall immune protection declines with age. Epigenomic analyses (e.g. snATACseq) will likely yield important insights into chromatin remodeling and could be informative in predicting how the cells will respond to stimulation. We also anticipate that alterations at the progenitor level underlie the effects we observed, and we suspect that increased levels of microbiome components and inflammatory cytokines in the circulation [6] induce the transcriptomic changes, which may be reinforced by epigenetic modifications.

Finally, we focused our study on classical monocytes, which in addition to yielding macrophages and moDC can also give rise to non-classical monocytes. It will thus be important to evaluate the heterogeneity and origins of non-classical monocytes too and define how they are impacted by aging. Collectively, these studies would provide key insights into the mechanisms underlying the aging-associated dysfunction of monocytes and other myeloid subsets derived from them.

## References

[1] Blacher E, et al. Nat Immunol. 2022; 23:229–36. 10.1038/s41590-021-01083-0 34949832 PMC9704320

[2] De Maeyer RP, Chambers ES. Immunol Lett. 2021; 230:1–10. 10.1016/j.imlet.2020.12.003 33309673

[3] Puchta A, et al. PLoS Pathog. 2016; 12:e1005368. 10.1371/journal.ppat.1005368 26766566 PMC4713203

[4] Yáñez A, et al. Immunity. 2017; 47:890–902.e4. 10.1016/j.immuni.2017.10.021 29166589 PMC5726802

[5] Barman PK, et al. Aging Cell. 2022; 21:e13701. 10.1111/acel.13701 36040389 PMC9577948

[6] Thevaranjan N, et al. Cell Host Microbe. 2017; 21:455–66.e4. 10.1016/j.chom.2017.03.002 28407483 PMC5392495

